# BK Channel Gating Mechanisms: Progresses Toward a Better Understanding of Variants Linked Neurological Diseases

**DOI:** 10.3389/fphys.2021.762175

**Published:** 2021-10-21

**Authors:** Jianmin Cui

**Affiliations:** Department of Biomedical Engineering, Center for the Investigation of Membrane Excitability Disorders, Cardiac Bioelectricity and Arrhythmia Center, Washington University, St. Louis, MO, United States

**Keywords:** BK channel, voltage, calcium, activation, neurological disorders, loss of function mutation, gain of function mutation

## Abstract

The large conductance Ca^2+^-activated potassium (BK) channel is activated by both membrane potential depolarization and intracellular Ca^2+^ with distinct mechanisms. Neural physiology is sensitive to the function of BK channels, which is shown by the discoveries of neurological disorders that are associated with BK channel mutations. This article reviews the molecular mechanisms of BK channel activation in response to voltage and Ca^2+^ binding, including the recent progress since the publication of the atomistic structure of the whole BK channel protein, and the neurological disorders associated with BK channel mutations. These results demonstrate the unique mechanisms of BK channel activation and that these mechanisms are important factors in linking BK channel mutations to neurological disorders.

## Introduction

BK channels are activated by membrane depolarization and intracellular Ca^2+^ binding. Due to its large single channel conductance of 100–300 pS (Latorre et al., 1989), which gives rise to its name as the big K^+^ (BK) channel, the opening of the channel effectively repolarizes the membrane and stops Ca^2+^ from entering the cell due to deactivation of voltage gated Ca^2+^ channels (Lancaster and Nicoll, 1987; Storm, 1987). Therefore, BK channels are important in controlling cellular excitation and Ca^2+^ homeostasis. In 2005, a mutation of the BK channel was found to associate with epilepsy and movement disorder in human patients (Du et al., 2005). The mutation in the *KCNMA1* gene that encodes the Slo1 α-subunit of BK channels causes a missense change, D434G. This mutation alters Ca^2+^ dependent activation of the channel, resulting in an enhanced Ca^2+^ sensitivity (Du et al., 2005; Díez-Sampedro et al., 2006; Lee and Cui, 2009; Yang et al., 2010). With the progress in human genetics, more *KCNMA1* variants that link to neurological disorders have been identified. Some of the mutations in BK channels due to these variants have been functionally characterized, and the results show that these mutations alter voltage and Ca^2+^ dependent activation to different effects (Bailey et al., 2019; Miller et al., 2021). These results demonstrate that the changes in voltage and Ca^2+^ dependent activation of BK channels are important factors linking the *KCNMA1* variants to neurological disorders.

Voltage and Ca^2+^ dependent activation of BK channels has been studied intensively since the discovery of BK channels in early 1980’s (Marty, 1981; Pallotta et al., 1981). During the course of these studies, the DNA sequence of Slo1 was identified (Atkinson et al., 1991; Adelman et al., 1992; Butler et al., 1993) and the atomistic structures of the channel protein were solved. The first atomistic structure data came from MthK, a K^+^ channel that lacks the voltage sensor but has a cytosolic structure resembling that of BK channels (Jiang et al., 2002a,b). The cytosolic structure of BK channels was subsequently solved using X-ray crystallography (Wu et al., 2010; Yuan et al., 2010; Yuan et al., 2012), and recently the structure of the whole BK channel with and without the association of the modulatory β4 subunit was solved using cryo-EM (Hite et al., 2017; Tao et al., 2017; Tao and MacKinnon, 2019). Each of these structural discoveries has revealed a new dimension in the organization of the molecular components important for voltage and Ca^2+^ dependent activation, allowed the use of additional approaches to explore the mechanisms, and as a result, led to a leap of understanding. In this article I will first review the studies prior to the publication of cryo-EM structures of BK channels, which have defined the important frameworks for understanding BK channel activation. The cryo-EM structures of the whole BK channel have revealed the interactions among structural domains with and without Ca^2+^ binding (Hite et al., 2017; Tao et al., 2017; Tao and MacKinnon, 2019) that are important for understanding the couplings of Ca^2+^ and voltage sensors to the opening of the pore. These structures also help reveal novel mechanisms of BK channel activation that differ from canonical mechanisms that were known in other K^+^ channels. Finally, the changes in voltage and Ca^2+^ dependent activation with some variants linked to neurological diseases will be described, and the relationship between the change of molecular mechanisms and clinical presentations will be discussed.

### Established Frameworks for Understanding BK Channel Activation

Ion channel activation involves three major molecular processes: sensor activation, sensor-pore coupling, and pore opening. Sensors in channel proteins change conformation upon the stimulation of signals, such as changes in the membrane potential and ligand binding. The conformational change of sensors is propagated to the pore *via* interactions between sensors and the pore, known as sensor-pore coupling. Finally, the pore opens to allow ionic flow across the membrane. BK channel activation depends on both membrane potential and intracellular Ca^2+^. The studies of BK channel activation have revealed the following frameworks for understanding how the two stimuli open the same pore.

**FIGURE 1:**
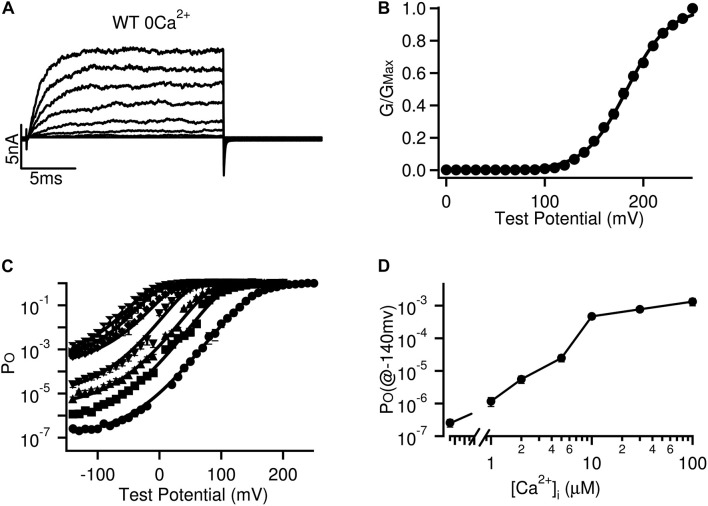
Independent voltage and Ca^2+^ activation of BK channels. **(A)** Current traces activated by voltage pulses in low Ca^2+^ concentration (≤0.5 nM) without binding to Ca^2+^. **(B)** Steady state conductance of the channels (measured at the end of the current traces in panel **(A)**) at different voltages. The GV relation reflects voltage dependence of open probability of the channels. **(C)** Open probability of BK channels at different voltage and Ca^2+^ concentrations. At negative voltages (V < −100 mV) channels open in response to Ca^2+^ concentration changes without dependence on voltage sensor activation. **(D)** Dependence of open probability at −140 mV on Ca^2+^ concentration. The same results have been published previously (Yang et al., 2010).

First, Ca^2+^ and voltage activate the channel with distinct mechanisms. In early studies the relation between Ca^2+^ and voltage in activating BK channels was an important question. The identification of Slo1 gene *KCNMA1* and the availability of cDNA of Slo1 for functional expression of BK channels in exogenous cells (Atkinson et al., 1991; Adelman et al., 1992; Butler et al., 1993) allowed the studies to distinguish the distinct Ca^2+^ and voltage dependent activation mechanisms (Cui et al., 1997). When the intracellular Ca^2+^ concentration was kept low (≤0.5 nM) BK channels opened in response to membrane depolarization with a rate that exceeded the diffusion limit for Ca^2+^ to bind, suggesting that the channel can open by voltage without Ca^2+^ binding (Cui et al., 1997; Figures 1A,B). On the other hand, when the voltage sensor was kept at the resting state by negative membrane potentials (≤−140 mV) (Horrigan et al., 1999; Cui and Aldrich, 2000) the open probability of BK channels increased by 4 orders of magnitude when Ca^2+^ concentration was elevated to 100 μM (Horrigan and Aldrich, 2002; Yang et al., 2010; Figures 1C,D), suggesting that the channel can open by Ca^2+^ binding without voltage. Subsequent studies identified the voltage sensor and Ca^2+^ binding sites in different structural domains of the channel. While the residues responsible for voltage sensing are located in the transmembrane segments (Diaz et al., 1998; Cui and Aldrich, 2000; Ma and Horrigan, 2005; Pantazis et al., 2010a; Zhang et al., 2014), the Ca^2+^ binding sites are found to reside in the cytosolic domain (Schreiber and Salkoff, 1997; Shi et al., 2002; Xia et al., 2002; Bao et al., 2004; Yusifov et al., 2008; Wu et al., 2010; Yuan et al., 2010; Zhang et al., 2010; Javaherian et al., 2011; Yuan et al., 2012; Hite et al., 2017; Tao et al., 2017; Tao and MacKinnon, 2019). Thus, voltage and Ca^2+^ activate the channel by perturbing different structure domains that harbor the respective sensors and can open the channel independent of each other.

**FIGURE 2:**
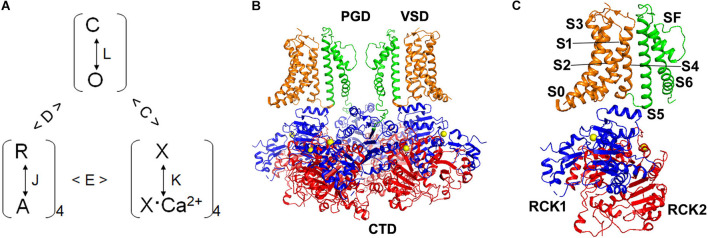
Allosteric activation mechanism and structure of BK channels. **(A)** The allosteric model of BK channel activation. The channel undergoes an intrinsic closed-open transition (C and O), which is regulated by voltage sensor activation (R to A) and Ca^2+^ binding (X to X:Ca^2+^) with allosteric mechanisms. i.e., with each voltage sensor activation the channel open state is favored by an allosteric factor D; and with each Ca^2+^ binding to the channel the channel open state is favored by an allosteric factor C. L, J, and K are equilibrium constants for the respective transitions. Although voltage and Ca^2+^ can activate the channel independently, voltage sensor activation and Ca^2+^ binding affect each other with an allosteric factor E. In this model only 4 Ca^2+^ binding sites are assumed. Similar models containing 8 Ca^2+^ binding sites can also fit the electrophysiology data (Savalli et al., 2012). **(B)** BK channel structure with four Slo1 subunits (PDB entry: 6V38). Only two subunits are shown for the membrane spanning part of the channel. VSD: voltage sensing domain, PGD: pore-gate domain, CTD: cytosolic domain. **(C)** A single Slo1 subunit (PDB entry: 6V38). S0–S6: transmembrane segments, SF: selectivity filter, the two Ca^2+^ bound to the channel are shown in yellow.

Second, Ca^2+^ and voltage activate the channel with allosteric mechanisms. In the extreme experimental conditions with low Ca^2+^ concentrations (≤0.5 nM) and negative membrane potentials (≤−140 mV) BK channels were observed to open with a small but measurable open probability (∼10^–7^) (Horrigan et al., 1999; Cui and Aldrich, 2000; Figure 1C). In these experimental conditions the channel opening was not controlled by Ca^2+^ binding or voltage sensor activation, but was an intrinsic spontaneous event. The open probability of BK channels increases with Ca^2+^ binding or voltage sensor activation, since at the open conformation the channel has a higher Ca^2+^ affinity and a facilitated voltage sensor activation (McManus and Magleby, 1991; Cox et al., 1997; Horrigan et al., 1999; Horrigan and Aldrich, 2002). Each BK channel contains eight high-affinity Ca^2+^ binding sites (Schreiber and Salkoff, 1997; Shi et al., 2002; Xia et al., 2002; Bao et al., 2004; Wu et al., 2010; Yuan et al., 2010, 2012; Zhang et al., 2010; Hite et al., 2017; Tao et al., 2017; Tao and MacKinnon, 2019) and four voltage sensors (Hite et al., 2017; Tao et al., 2017; Tao and MacKinnon, 2019; Figure 2), and the open probability of the closed-open transitions increases with each Ca^2+^ binding and voltage sensor activation. This mechanism of Ca^2+^ and voltage dependent activation can be quantitatively described by a model that accounts for BK channel activation with the changes of Ca^2+^ concentration and voltage at a broad range that includes and extends beyond physiological conditions (Horrigan and Aldrich, 2002; Figure 2A). This mechanism is in contrast to Shaker K^+^ channels, for which the opening was tightly controlled by voltage sensor activation even when the open probability was at 10^–7^ (Islas and Sigworth, 1999). Thus, the mechanism of coupling between sensor activation and pore opening in Shaker K^+^ channels is thought to be obligatory, while in BK channels is allosteric. The allosteric mechanism makes it possible for BK channels to sense both Ca^2+^ binding and voltage sensor activation for opening. In a channel with the pore tightly controlled by the voltage sensor, such as Shaker, Ca^2+^ binding would not have been able to open the channel without voltage sensor activation.

Third, interactions among structural domains are important for Ca^2+^ and voltage dependent activation of BK channels (Figure 2B). The primary sequence of Slo1 channels, in comparison with voltage gated K^+^ (Kv) channels, indicated that the channel included three structural domains, the transmembrane pore-gate domain (PGD), the transmembrane voltage sensor domain (VSD), and the cytosolic domain (CTD) (Cui, 2010; Lee and Cui, 2010). The structure of MthK (Jiang et al., 2002a,b) and subsequently the structures of the BK CTD (Wu et al., 2010; Yuan et al., 2010; Yuan et al., 2012) showed that the CTD contains two RCK (Regulator of K^+^ Conductance) domains in each Slo1 subunit, and the eight RCK domains of the four subunits form a ring-like structure called the gating ring. Based on the sequence homology with Kv channels, the structures of the VSD, which contains the transmembrane segments S1–S4, and PGD, which contains S5–S6, were modeled after the structure of Kv1.2 (Long et al., 2005a; Lee and Cui, 2009). Two Ca^2+^ binding sites, the Ca^2+^ bowl that is primarily formed by the RCK2 residues (Schreiber and Salkoff, 1997; Bao et al., 2004; Yusifov et al., 2008; Wu et al., 2010; Hite et al., 2017; Tao et al., 2017; Tao and MacKinnon, 2019) and the site that is located in RCK1 (Shi et al., 2002; Xia et al., 2002; Wu et al., 2010; Zhang et al., 2010; Yuan et al., 2012; Hite et al., 2017; Tao et al., 2017; Tao and MacKinnon, 2019), were found in the CTD of each Slo1. Residues in the VSD that are important for voltage sensing were identified (Ma and Horrigan, 2005; Zhang et al., 2014). Thus, the VSD and CTD harbor the voltage and Ca^2+^ sensors and propagate the stimuli to open the pore in PGD *via* interactions among these structure domains.

### Domain-Domain Interactions for Sensor-Pore Couplings in BK Channel Activation

Prior to the publication of the cryo-EM structures of the whole BK channel (Hite et al., 2017; Tao et al., 2017; Tao and MacKinnon, 2019; Figures 2B,C) the coupling of Ca^2+^ binding in the CTD to the opening of the PGD had been proposed to involve two types of interactions among the structure domains. A study Niu et al. (2004) showed that voltage and Ca^2+^ dependent activation of BK channels was changed by altering the covalent link between the PGD and the CTD *via* a peptide of 15 residues, known as the C-linker. The changes in activation depended on the changes in C-linker length by addition or deletion of amino acids in a relationship as if the interaction between the PGD and CTD were like pulling a spring for channel activation. Thus, it was proposed that Ca^2+^ may activate the channel by causing a conformational change in the CTD that directly tugs the pore to open *via* the C-linker. On the other hand, the CTD was proposed to also interact with the VSD *via* non-covalent interactions, and such interactions may indirectly open the pore *via* VSD-PGD interactions (Lee and Cui, 2009). This proposal derived from the finding that the residues from both the VSD and CTD form a Mg^2+^ binding site that modulates voltage dependent activation (Shi and Cui, 2001; Hu et al., 2003; Zeng et al., 2005; Yang et al., 2007; Yang et al., 2008), and thus VSD and CTD are located closely. This mechanism was consistent with an earlier finding that the part of the CTD that is located close to the VSD is important in determining the different Ca^2+^ sensitivities between different BK channel homologs (Krishnamoorthy et al., 2005), and subsequent studies showed that alterations of the VSD-CTD interactions indeed affected Ca^2+^ dependent activation (Yang et al., 2013; Geng et al., 2020).

For voltage dependent activation, it was found that a negatively charged residue E219 in the VSD, which contributed to voltage sensing, interacted with charged residues E321 and E324 in the PGD to mediate the coupling of voltage sensor activation to channel opening (Zhang et al., 2014). The interaction, however, was also found to be involved in the coupling between Ca^2+^ bindings and pore opening. Similar to Ca^2+^ dependent activation that involves interactions among all CTD, VSD, and PGD, voltage dependent activation also involves all three structure domains. A comparison between the voltage dependent activation of the wild type BK channel with that of a truncated BK channel with CTD deletion (Budelli et al., 2013) showed that the CTD was important in the coupling between VSD activation and pore opening (Zhang et al., 2017). The CTD undergoes conformational changes during voltage dependent activation as detected by fluorescence signals (Miranda et al., 2018). Ca^2+^ binding to the CTD also alters voltage dependence of channel activation (Horrigan and Aldrich, 2002; Sweet and Cox, 2008; Savalli et al., 2012; Lorenzo-Ceballos et al., 2019). These results suggest that the interactions among all three structure domains are involved in voltage and Ca^2+^ dependent activation.

The CTD-VSD non-covalent interactions were more clearly shown by the cryo-EM structures of the whole BK channels (Hite et al., 2017; Tao et al., 2017; Tao and MacKinnon, 2019; Figures 2B,C). The CTD gating ring is located closely to the membrane spanning domains of the channel, with an extensive interface between the N-terminus of the CTD with the cytosolic part of the VSD (585 Å^2^ per subunit in the Ca^2+^ structure) (Hite et al., 2017). Comparing the structure with Ca^2+^/Mg^2+^ bound to the metal-free structure the N-lobes of the RCK1 domain, which face the VSD, tilted in a rigid body fashion away from the pore axis. In the Ca^2+^/Mg^2+^ bound structure the cytosolic part of the VSD also showed a corresponding outward displacement, and the movements of both the RCK1 and VSD alter the interface between these domains. This Ca^2+^ dependent change in the interactions between CTD and VSD suggests that these interactions are part of the coupling mechanism for Ca^2+^ binding to open the channel, and is consistent with the studies to show that mutations in the VSD-CTD interface alter Ca^2+^ sensitivity (Yang et al., 2013; Geng et al., 2020). The C-linker in the cryo-EM structures is partly α-helical and partly extended and nearly identical in both the structures with and without Ca^2+^/Mg^2+^ bound, but undergoes a large positional displacement laterally as a rigid unit. Recent studies showed that the C-linker can interact with the membrane directly to alter BK channel activation (Tian et al., 2019; Yazdani et al., 2020b). These results suggest that the C-linker may not affect channel activation simply as a passive link between the CTD and PGD, but directly interact with the membrane and other parts of the channel protein such as the gating ring to mediate Ca^2+^ and voltage dependent activation.

In the tetrameric structure of the Kv1.2 channel the VSD of each subunit is adjacent to the PGD of its neighboring subunit, showing a domain-swapped configuration (Long et al., 2005b). The BK channel, however, does not show such a domain-swapping. Instead, the VSD and PGD of the Slo1 subunit interact within each subunit (Hite et al., 2017; Tao et al., 2017; Tao and MacKinnon, 2019; Figure 2C). This difference as revealed by the cryo-EM structure of BK channels suggests that the coupling between VSD activation to pore opening differs from the canonical electromechanical coupling mechanism for the domain-swapped Kv channels. In domain swapped Kv channels the peptide linking the VSD and the PGD of a subunit, the S4–S5 linker, interacts with the cytosolic part of the transmembrane helix S6 (Long et al., 2005b). S6 lines the inner pore, and the helices from four subunits cross at the cytosolic side of the membrane to form the activation gate that controls ionic flow during the opening and closing of these Kv channels (Liu et al., 1997; Long et al., 2005a,b). The interactions between the S4–S5 linker and S6 has been shown to mediate electromechanical coupling, which open the activation gate in response to VSD movements (Lu et al., 2001; Long et al., 2005b; Hou et al., 2020; Cowgill and Chanda, 2021). However, in BK channels, the S4–S5 linker of each subunit is short and not located close to S6, but interacts with the N-terminus of RCK1 (Hite et al., 2017; Tao et al., 2017; Tao and MacKinnon, 2019; Figure 2C). The VSD seems to be in close contact with the PGD within the same Slo1 subunit only with the interface between S4 and S5 helices (Figures 2B,C). The contact between S4 and S5 helices is extensive, which suggests that the interactions between the two helices are important for mediating the coupling between VSD activation and pore opening.

The extensive interactions between the S4 and S5 would also restrict the motion of S4, which may be responsible for the unique VSD movements during BK channel activation. In domain swapped Kv channels the charged residues that sense voltage for channel activation, known as gating charges, are primarily found in the S4 transmembrane helix (Aggarwal and MacKinnon, 1996; Mannuzzu et al., 1996; Seoh et al., 1996). S4 moves across the membrane with a large distance upon membrane potential depolarization, resulting in a transfer of total 12–16 equivalent gating charges across the electric filed in the membrane (Zagotta et al., 1994a,b; Aggarwal and MacKinnon, 1996; Mannuzzu et al., 1996; Seoh et al., 1996; Bezanilla, 2000; Gandhi and Isacoff, 2002). However, in BK channels gating charges are found in S2 (D153 and R167), S3 (D186), and S4 (R213, E219) segments (Ma and Horrigan, 2005; Pantazis et al., 2010a; Zhang et al., 2014). During voltage dependent activation of BK channels a total 2.32 equivalent gating charges are moved across the electric filed in the membrane (Horrigan and Aldrich, 1999; Ma and Horrigan, 2005), much smaller as compared to those in domain swapped Kv channels. These results suggest that the VSD may undergo small movements that involve many transmembrane segments during voltage dependent activation (Ma and Horrigan, 2005; Pantazis et al., 2010a,b; Pantazis and Olcese, 2012; Pantazis et al., 2018). Furthermore, the pore opening in BK channels also retrospectively control VSD activation, such that VSD activation increases with multiple time courses, and some of which are in parallel with channel opening (Horrigan and Aldrich, 1999; Savalli et al., 2006), consistent with the idea that the S4–S5 interactions may restrict VSD movements during BK channel activation.

### Hydrophobic Gate in BK Channels

Cryo-EM structures of BK channels reveal that the S6 helices from the four Slo1 subunits do not cross at the cytosolic side, either with or without Ca^2+^/Mg^2+^ bound (Figure 2B). If the metal-free structure represents the closed state of the channel, while the Ca^2+^/Mg^2+^ bound structure represents an open state (Hite et al., 2017), the cytosolic side of the pore is thus wide open at both the open and closed states of the channel. This result is consistent with the suggestion from the studies of BK channel block by quaternary ammonium (QA) molecules (Li and Aldrich, 2004; Wilkens and Aldrich, 2006; Tang et al., 2009) and other smaller molecules (Zhou et al., 2011; Zhou et al., 2015) that the channels do not have an intracellular gate so that the blockers can enter the pore even at the closed state. The study of a peptide blocker suggests that the cytosolic part of the pore may undergo a conformational change to reduce the pore size during BK channel closing (Li and Aldrich, 2006), but the change is not sufficient to close the pore to restrict the entrance of K^+^ ions or QA blockers. These results indicate that the activation gate of BK channels may be located above the cytosolic side, possibly at the selectivity filter, which is the narrowest part of the pore (Figures 2B,C) and with a conserved function among K^+^ channels to select K^+^ ion over other ions for permeation (Hite et al., 2017; Tao et al., 2017; Tao and MacKinnon, 2019).

Recently, an alternative mechanism for the activation gate was proposed. The structures of the metal free and Ca^2+^/Mg^2+^ bound BK channels suggested that the inner pore of the channel underwent a conformational change in the absence of metal binding, which mainly involved an amphipathic segment of S6, V_319_PEIIE_324_, to expose hydrophobic residues V319 and I323 to the pore inner surface (Hite et al., 2017; Tao et al., 2017; Tao and MacKinnon, 2019). As a result of these changes the inner pore beneath the selectivity filter becomes narrower, more elongated and hydrophobic. A molecular dynamics simulation study found that these changes promote dewetting transitions that completely deplete the inner pore of liquid water, giving rise to a vapor barrier to block the ion flow (Jia et al., 2018). Importantly, the dry pore remains physically open with an average diameter of ∼6 Å, allowing QA blockers to access the deep-pore region to block the channel even in the closed state. Such a hydrophobic gate depends on the hydrophobicity of the surface residues and the geometry of the inner pore (Aryal et al., 2015; Yazdani et al., 2020a). A functional study showed that the mutations in the BK channel inner pore A316D and A316V made the channel constitutively open and harder to open, respectively (Chen et al., 2014). Consistent with these results molecular dynamics simulations found that A316D reduced hydrophobicity of the pore and prevented dewetting transitions, while A316V enhanced hydrophobicity of the pore and made dewetting transitions faster with less water molecules remaining in the pore (Jia et al., 2018).

At present the hydrophobic gating mechanism is primarily based on molecular dynamic simulations, which can explain the experimental results of QA blockers on BK channel and some mutations. There has not been a direct experimental validation of this mechanism, partly due to the dilemma that once the vapor barrier forms the channel is closed and devoid of any functional detection. Comparing to the hydrophobic gate, the alternative mechanism, the selectivity filter acting as the activation gate, is not clearly defined with computational or experimental evidence. Where is the activation gate of BK channels and how it operates are not only part of the fundamental mechanism of voltage and Ca^2+^ dependent activation but also important for understanding BK channel modulation by compounds that act in the pore of BK channels, such as the QA blockers, paxilline (Zhou et al., 2020), and NS11021 (Rockman et al., 2020; Schewe et al., 2019). Some of these modulators have been excellent tools for the studies of the roles of BK channels in cellular and tissue function (Kaczorowski and Garcia, 2016; Cui, 2020), and may serve as the lead for therapies for BK variants linked neurological diseases as described in the following sections.

### Slo1 Mutation D434G Enhances Ca^2+^ Sensitivity and Is Associated With Epilepsy and Paroxysmal Non-kinesigenic Dyskinesia

**FIGURE 3:**
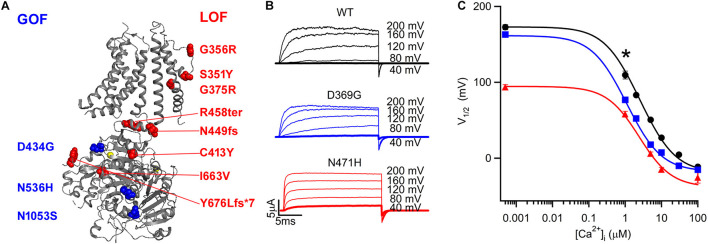
BK mutations alter activation and link to neurological diseases. **(A)** The GOF (blue) and LOF (red) mutations mapped onto Slo1 structure. Yellow circles: Ca^2+^ ions bound to the channel. **(B)** The GOF mutation D434G and N536H in human Slo1 corresponds to D369G and N471H in mouse Slo1, respectively. Both mutations enhance currents at the same voltage and Ca^2+^ concentration. The current trace at +40 mV, which is most relevant physiological membrane potential, is highlighted in a thicker line. **(C)** Ca^2+^ dependence of the V_1/2_ of GV relations (the voltage where GV relation is half maximum, which is used to measure the voltage range in which the channel activates). The result shows that with the D369G and N471H mutations GV shifts to more negative voltages at all Ca^2+^ concentrations. The * shows that 1 μM Ca^2+^ concentration is most physiologically relevant. The same results have been published previously (Yang et al., 2010; Zhang et al., 2020).

In a study of a family with inherited generalized epilepsy and coexistent paroxysmal dyskinesia (GEPD), a heterozygous A-G transition in exon 10 of *KCNMA1* was identified, which results in the D434G mutation in the BK channel subunit Slo1 (Du et al., 2005; Figure 3A). The inheritance followed an autosomal dominant pattern, and sixteen members of the family were affected. Among these individuals nine had early childhood onset of absence epilepsy, showing the characteristic episodes of loss of awareness, with vacant staring and unresponsiveness, and electroencephalography (EEG) showing synchronous spike-and-wave discharges (SWDs). Three of these individuals also developed generalized tonic-clonic seizures. The seizures in some of these individuals were responsive to anti-epilepsy medicine valproate, lamotrigine, or clonazepam. Among the sixteen affected individuals twelve had paroxysmal dyskinesia including five with both seizures and paroxysmal dyskinesia. These individuals were described to have involuntary dystonic or choreiform movements of the mouth, tongue, and extremities. The episodes were not induced by sudden movements, but induced by alcohol, fatigue, and stress, so that the paroxysmal dyskinesia was classified as paroxysmal non-kinesigenic dyskinesia (PNKD). It was noted that the episodes of PNKD developed around the same age as the onset of the seizures, but during the episodes the individual had preserved consciousness.

Comparison of the currents of the D434G BK channels with the wild type (WT) BK currents showed that the D434G currents were increased with a faster activation time course at the same voltage and intracellular Ca^2+^ concentrations ≥1 μM (Du et al., 2005; Díez-Sampedro et al., 2006; Lee and Cui, 2009; Wang et al., 2009; Yang et al., 2010; Moldenhauer et al., 2020), which is the cytosolic Ca^2+^ concentration at neuronal excitations (Figures 3B,C). These results suggest that more currents flow through the D434G BK channels during an action potential. At these Ca^2+^ concentrations, the voltage dependence of channel conductance (GV relation) of D434G BK channels was shifted to more negative voltages (Figure 3C), indicating that channel activation was increased. The increased channel activation was actually primarily because of an increased Ca^2+^ sensitivity without changes in voltage dependent activation, because the mutation increased the apparent affinity for Ca^2+^ in channel activation (Díez-Sampedro et al., 2006; Wang et al., 2009; Yang et al., 2010), while at low Ca^2+^ concentrations (∼0.5 nM) with few Ca^2+^ binding to the channel the D434G mutation did not alter GV relation (Díez-Sampedro et al., 2006; Yang et al., 2010). The residue D434 is located close to the Ca^2+^ binding site in RCK1 (Figure 3A). The mutation D434G neutralizes a negative charge close to the Ca^2+^ binding site and makes the protein structure more flexible around the site, which may be responsible for the mutation to specifically enhance the affinity of Ca^2+^ for the RCK1 site and strengthen the coupling between Ca^2+^ binding to the site with pore opening (Yang et al., 2010).

Recently a knock-in mouse model carrying BK D434G mutation was characterized (Dong et al., 2021). The BK D434G mutation mice manifested symptoms resembling the human patients affected by the BK D434G mutation. Simultaneous video-EEG recordings showed that the BK-D434G mice had frequent episodes of spontaneous, generalized SWDs, and during which behavioral arrests, characteristic of absence seizures. These mice were also more susceptible to the induction of generalized seizures by injection of low dose of pentylenetetrazole (PTZ), a convulsant, than the WT mice, consistent with that some BK-D434G patients developed generalized tonic-clonic seizures (Du et al., 2005). Consistent with the human patients, the mutant animals exhibited severe locomotive defects in a battery of motor tests when there was no absence seizure. However, due to the limitations of mouse models, it is difficult to clearly tell if the animals also had PNKD. Both the heterozygous BK-D434G mutation (BK^*DG/WT*^) and the homozygous mutation (BK^*DG/DG*^) mice exhibited these symptoms, but the incidents were more frequent or severe in BK^*DG/DG*^ mice. All these symptoms could be suppressed by the treatment with a BK channel inhibitor, paxilline (PAX). These results support the idea that the D434G mutation in BK channels causes a gain of function of the channel that results in epilepsy and movement disorders.

Acute brain slice recordings from the BK D434G mutation mice further showed that the mutation caused hyperexcitability in both the cortical pyramidal neurons and the cerebellar Purkinje cells (Dong et al., 2021), which play essential roles in the pathogenesis of absence seizures (Crunelli et al., 2020) and contribute to the motor defects (Sausbier et al., 2004), respectively. These neurons from the BK^*DG/WT*^ mice exhibited a significantly increased action potential frequency compared with the BK^*WT/WT*^ mice. The action potentials in BK^*DG/WT*^ neurons showed a much faster repolarization with a shortened duration and augmented after-hyperpolarization amplitude (AHP). These results are consistent with that the BK-D434G channels have a higher Ca^2+^ sensitivity (Díez-Sampedro et al., 2006; Wang et al., 2009; Yang et al., 2010), allowing the channels to activate more during an action potential following membrane depolarization and the opening of voltage gated Ca^2+^ channels. The enhanced BK currents more efficiently hyperpolarize the membrane, enabling a faster recovery of the voltage-gated sodium channels from inactivation and potentially facilitating the activation of the hyperpolarization-activated cation (HCN) channels (Lancaster and Nicoll, 1987; Storm, 1987; Shah, 2014), which allow the neurons fire at a higher frequency. The application of BK channel inhibitor PAX widened the action potential, reduced AHP and decreased firing frequency.

The seizures in patients affected by BK D434G were responsive to general anti-epilepsy medicine (Du et al., 2005), and so were in the BK D434G mutation mice (Dong et al., 2021). Both ethosuximide and valproate suppressed generalized SWDs (Dong et al., 2021). It was shown that the anti-epilepsy medicine acetazolamide had no direct effect on BK channels (Moldenhauer et al., 2020), suggesting that BK channels were not the target of these general anti-epilepsy medicines. It is reported that 30% of absence epilepsy patients are pharmaco-resistant with the first-line anti-absence medicines (Crunelli et al., 2020). The results of BK channel inhibitor PAX (Dong et al., 2021) suggest that BK channels as a new target for anti-absence therapy may be developed to treat those pharmaco-resistant patients. However, the effects of PAX in suppressing generalized SWDs in BK D434G mutation mice only lasted for ∼30 min, which indicates that novel BK channel inhibitors with appropriate efficacy, specificity and pharmacokinetics need to be developed. The development of such novel drugs will benefit from the understanding of molecular mechanisms of BK channel gating.

### Neurological Diseases Associated With Other BK Channel Gain of Function Mutations

The second disease associated *KCNMA1* variant was identified in 2015, which resulted in the N1053S mutation in Slo1 (Zhang et al., 2015; Figure 3A). Subsequently more patients with the same Slo1 mutation were identified [also called N995S (Li et al., 2018), or N999S (Heim et al., 2020)]. These mutations were all *de novo* in unrelated patients. Some of these patients suffered movement disorders, diagnosed with PNKD (Zhang et al., 2015) or cataplexy (Heim et al., 2020), while some only suffered absence epilepsy or mixed with myoclonic seizures (Li et al., 2018). Only one patient suffered both symptoms (Heim et al., 2020). These patients also showed various degrees of developmental delay or intellectual disability. Whether symptoms respond to anti-epileptic medicines also varied among these patients.

As the symptoms of BK-N1053S patients showed some resemblance to those of BK-D434G patients, N1053S mutation is also a gain of function (GOF) mutation. Similar to D434G, the N1053S mutation increased BK currents and the activation kinetics at physiological intracellular Ca^2+^ concentrations with all depolarizing voltages (Li et al., 2018; Moldenhauer et al., 2020), suggesting that the mutation in neurons would shorten action potentials, increase after hyperpolarization and enhance spike firing frequencies (Li et al., 2018). However, the molecular mechanism of N1053S in causing functional changes differs from that of D434G. While G434G primarily enhances Ca^2+^ sensitivity, N1053S does not seem to alter Ca^2+^ dependent activation. The G-V relation of the N1053S BK channels shifts to negative voltages with a similar amount at different Ca^2+^ concentrations, and the deletion of Ca^2+^ binding sites does not prevent the G-V shift of the N1053S BK channels (Li et al., 2018).

Recently another *de novo* GOF *KCNMA1* variant that resulted in a Slo1 N536H mutation (Figure 3A) was identified, with which the patient suffered frequent dystonic/atonic spells (Zhang et al., 2020). She also had disorders of autism spectrum, attention deficit hyperactivity, and intellectual disability. No obvious seizure was observed in the patients. The symptoms were not responsive to antiepileptic medicines, but dextroamphetamine, a central nervous system stimulant, completely controlled her dystonic episodes from >100 per day to none. N536H is also a GOF mutation that increases BK currents at 1 μM Ca^2+^ concentration by shifting G-V relation to negative voltages (Figures 3B,C). Similar to N1053S, but unlike G434G, N536H does not alter Ca^2+^ dependent activation of the BK channel.

The findings so far suggest that the BK channel GOF mutations are associated with absence epilepsy with possible development of myoclonic seizures, movement disorders, or both symptoms. However, it needs to be emphasized that these symptoms vary from patient to patient. The patients may also have cerebellar atrophy, development delays, autism spectrum and intellectual disability to various degrees. These different symptoms derive from increased BK currents either due to increased Ca^2+^ sensitivity or shift of G-V relation with an intact Ca^2+^ dependent activation. The larger BK currents may result in hyperactivity in different neuron types to cause various symptoms. Future studies are needed to dissect how the GOF BK channel mutations with increased Ca^2+^ sensitivity or shift of G-V relation with an intact Ca^2+^ dependent activation lead to different neurological symptoms.

### Neurological Diseases Associated With BK Channel Loss of Function Mutations

A report in Tabarki et al. (2016) described two sisters harboring a homozygous *KCNMA1* variant that resulted in the frameshift mutation T676Lfs^∗^7, which changed T676 to L and caused a frame shift after 7 residues downstream (Figure 3A). The patients suffered development delays, severe cerebellar atrophy and seizures of myoclonic type, which progressed to tonic seizures with one of the siblings. The homozygous frameshift mutation presumably abolished the BK channel function although no functional studies were conducted. Several other loss of function (LOF) *de novo* BK channel mutations that either completely destroyed BK channel function or reduce BK channel currents were subsequently identified in unrelated patients. The mutations that destroyed BK channel function included G375R, S351Y, G356R, N449fs, I663V (Liang et al., 2019) and the truncation mutation R458ter (Yesil et al., 2018). Other mutations, including C413Y and P805L, decrease BK currents either by reduced protein expression or a shift of the G-V relation to the positive voltages (Liang et al., 2019; Figure 3A). These patients suffered neurological diseases that vary in symptoms and severity, most had development delay, intellectual disability, movements disorders (such as ataxia and axial hypotonia), and cerebellar atrophy. Some of the patients also had seizures.

These results reveal that the normal neurological functions are sensitive to BK channel function. Either a GOF or LOF BK channel mutation could tip the balance of neural function and lead to neurological diseases. While the symptoms of both GOF and LOF BK channel mutations show some overlap, such as development delay, intellectual disability, movement disorder, and epilepsy, the LOF BK channel mutations result in more severe cerebellar atrophy. The patients with BK G375R mutation even had visceral and cardiac malformations, connective tissue symptoms, and dysmorphic features, suggesting that BK channel mutations can impact organs beyond the nervous system (Liang et al., 2019). In a recent study a BK LOF mutation, G354S (Figure 3A), was identified in association with cerebellar degeneration, ataxia, developmental delay and intellectual disability in a young girl (Du et al., 2020). The mutation caused a shift of G-V relation of BK channel activation to more negative voltages. However, due to its location in the selectivity filter of the channel the G354S mutation reduced single channel conductance and increased Na^+^ permeability, thereby decreasing the macroscopic currents and making the currents less effective in repolarizing the membrane potential. Viral transfection of the G354S BK in mouse brain induced ataxia in the animals. Transfection of the G354S BK into dividing PC12 cells, the mutant channel suppressed outgrowth of neurites by reducing the neurite length. The mutant BK channel was also toxic to mitochondria, with the cells expressing the G354S BK showing reduced mitochondria content, disrupted mitochondria superstructure, altered mitochondria dynamics to increase fragmented fission forms, and decreased mitochondria membrane potential. NS1619, a BK channel activator, protected the cells expressing G354S BK from the reduced neurite outgrowth, cell death, and changes in mitochondria, suggesting that the recovery of BK channel function can be a therapy for the neurological diseases associated with BK channel LOF mutations.

## Concluding Remarks

In ion channel activation sensors change conformation upon physical or chemical stimulation, which induces pore opening *via* interactions between sensors and the pore known as sensor-pore coupling. BK channel activation has shown unique characteristics in all these molecular steps as compared to canonical activation mechanisms in voltage gated K^+^ (Kv) channels. First, BK channels sense both membrane voltage and intracellular Ca^2+^ with distinct voltage sensor and Ca^2+^ binding sites. For these two stimuli to control the opening of the same pore independently the sensors are coupled with the pore with allosteric mechanisms. Second, the VSD and PGD domains in BK channels are not domain swapped among subunits, which differs from most Kv channels, and thus the VSD-pore coupling mechanism in BK channels is also unique. Third, the activation gate in BK channels is not a physical barrier at the intracellular side of the pore, but may be a vaper barrier as in a hydrophobic gating mechanism.

Mutations of BK channels that either increase BK channel currents (GOF) or decrease currents (LOF) at physiological voltages and Ca^2+^ concentrations are associated with neurological disorders. The changes in BK channel currents alter excitability of various neurons that may induce the symptoms. The LOF BK mutations may also directly disrupt mitochondria functions and cause cell death in brain and other organs. In the future, it is critical to translate the comprehensive understanding of BK channel structure and function to uncover the neurological mechanisms of BK channelopathy and design precision therapies to treat these patients. A key question is how the mutations of BK channels that either increase BK channel currents (GOF) or decrease currents (LOF) at physiological voltages and Ca^2+^ concentrations are associated with such a wide spectrum of neurological disorders. Animal models carrying different BK channel mutations and induced pluripotent stem cells (iPSCs) directly derived from patients are invaluable tools to address this question.

On the other hand, the change of voltage and Ca^2+^ dependent activation by the disease-associating mutations also provide unique insights for further understanding of BK molecular mechanisms. For instance, while we can reason that G375R (Liang et al., 2019; Figure 3A) may disrupt the opening of the activation gate since this is part of the diglycine hinge for the BK channel activation gate (Magidovich and Yifrach, 2004) and G356R may destroy the selectivity filter (Tao and MacKinnon, 2019; Figure 3A), we have no clue how the mutations in the cytosolic domain, such as N1053S (Zhang et al., 2015), N536H (Zhang et al., 2020), C413Y, or I663V (Liang et al., 2019; Figure 3A) may affect voltage dependent activation or the intrinsic gate opening in BK channels. Further studies of these mutations may provide insights to how the CTD interacts with VSD and PGD to control BK channel activation.

The studies of human genetics on BK channelopathy, biophysical characterizations, and the animal models carrying either GOF or LOF BK channel mutations indicate that BK channels can be a promising drug target for treating associated neurological diseases (Du et al., 2020; Dong et al., 2021). The studies of molecular mechanisms of BK channel activation and neurological diseases associated with aberrant BK channel function are valuable in directing the diagnoses of such diseases and the development of BK channel specific modulators for the therapy.

## Author Contributions

The author confirms being the sole contributor of this work and has approved it for publication.

## Conflict of Interest

The author declares that the research was conducted in the absence of any commercial or financial relationships that could be construed as a potential conflict of interest. The reviewer YZ declared a shared affiliation, with no collaboration, with one of the authors JC to the handling editor at the time of the review.

## Publisher’s Note

All claims expressed in this article are solely those of the authors and do not necessarily represent those of their affiliated organizations, or those of the publisher, the editors and the reviewers. Any product that may be evaluated in this article, or claim that may be made by its manufacturer, is not guaranteed or endorsed by the publisher.

## References

[B1] Adelman J. P. Shen K. Z. Kavanaugh M. P. Warren R. A. Wu Y. N. Lagrutta A. (1992). Calcium-activated potassium channels expressed from cloned complementary DNAs. *Neuron* 9 209–216. 10.1016/0896-6273(92)90160-F 1497890

[B2] Aggarwal S. K. MacKinnon R. (1996). Contribution of the S4 segment to gating charge in the Shaker K+ channel. *Neuron* 16 1169–1177. 10.1016/S0896-6273(00)80143-9 8663993

[B3] Aryal P. Sansom M. S. P. Tucker S. J. (2015). Hydrophobic gating in ion channels. *J. Mol. Biol.* 427 121–130. 10.1016/j.jmb.2014.07.030 25106689 PMC4817205

[B4] Atkinson N. S. Robertson G. A. Ganetzky B. (1991). A component of calcium-activated potassium channels encoded by the *Drosophila* slo locus. *Science* 253 551–555. 10.1126/science.1857984 1857984

[B5] Bailey C. S. Moldenhauer H. J. Park S. M. Keros S. Meredith A. L. (2019). KCNMA1-linked channelopathy. *J. Gen. Physiol.* 151 1173–1189. 10.1085/jgp.201912457 31427379 PMC6785733

[B6] Bao L. Kaldany C. Holmstrand E. C. Cox D. H. (2004). Mapping the BKCa channel’s “Ca2+ bowl”: side-chains essential for Ca2+ sensing. *J. Gen. Physiol.* 123 475–489. 10.1085/jgp.200409052 15111643 PMC2234491

[B7] Bezanilla F. (2000). The voltage sensor in voltage-dependent ion channels. *Physiol. Rev.* 80 555–592. 10.1152/physrev.2000.80.2.555 10747201

[B8] Budelli G. Geng Y. Y. Butler A. Magleby K. L. Salkoff L. (2013). Properties of Slo1 K+ channels with and without the gating ring. *Proc. Natl. Acad. Sci. U.S.A.* 110 16657–16662. 10.1073/pnas.1313433110 24067659 PMC3799338

[B9] Butler A. Tsunoda S. McCobb D. P. Wei A. Salkoff L. (1993). mSlo, a complex mouse gene encoding “maxi” calcium-activated potassium channels. *Science* 261 221–224. 10.1126/science.7687074 7687074

[B10] Chen X. Yan J. Aldrich R. W. (2014). BK channel opening involves side-chain reorientation of multiple deep-pore residues. *Proc. Natl. Acad. Sci. U.S.A.* 111 E79–E88. 10.1073/pnas.1321697111 24367115 PMC3890798

[B11] Cowgill J. Chanda B. (2021). Mapping electromechanical coupling pathways in voltage-gated ion channels: challenges and the way forward. *J. Mol. Biol.* 433:167104. 10.1016/j.jmb.2021.167104 34139217 PMC8579740

[B12] Cox D. H. Cui J. Aldrich R. W. (1997). Allosteric gating of a large conductance Ca-activated K+ channel. *J. Gen. Physiol.* 110 257–281. 10.1085/jgp.110.3.257 9276753 PMC2229366

[B13] Crunelli V. Lőrincz M. L. McCafferty C. Lambert R. C. Leresche N. Di Giovanni G. (2020). Clinical and experimental insight into pathophysiology, comorbidity and therapy of absence seizures. *Brain* 143 2341–2368. 10.1093/brain/awaa072 32437558 PMC7447525

[B14] Cui J. (2010). BK-type calcium-activated potassium channels: coupling of metal ions and voltage sensing. *J. Physiol.* 588 4651–4658. 10.1113/jphysiol.2010.194514 20660558 PMC3010134

[B15] Cui J. (2020). The action of a BK channel opener. *J. Gen. Physiol.* 152:e202012571. 10.1085/jgp.202012571 32342095 PMC7266148

[B16] Cui J. Aldrich R. W. (2000). Allosteric linkage between voltage and Ca2+-dependent activation of BK-type mslo1 K+ channels. *Biochemistry* 39 15612–15619. 10.1021/bi001509+ 11112549

[B17] Cui J. Cox D. H. Aldrich R. W. (1997). Intrinsic voltage dependence and Ca2+ regulation of mslo large conductance Ca-activated K+ channels. *J. Gen. Physiol.* 109 647–673. 10.1085/jgp.109.5.647 9154910 PMC2217061

[B18] Diaz L. Meera P. Amigo J. Stefani E. Alvarez O. Toro L. (1998). Role of the S4 segment in a voltage-dependent calcium-sensitive potassium (hSlo) channel. *J. Biol. Chem.* 273 32430–32436. 10.1074/jbc.273.49.32430 9829973

[B19] Díez-Sampedro A. Silverman W. R. Bautista J. F. Richerson G. B. (2006). Mechanism of increased open probability by a mutation of the BK channel. *J. Neurophysiol.* 96 1507–1516. 10.1152/jn.00461.2006 16738211

[B20] Dong P. Zhang Y. Mikati M. A. Cui J. Yang H. (2021). Neuronal mechanism of a BK channelopathy in absence epilepsy and movement disorders. *bioRxiv* [Preprint] bioRxiv: 2021.06.30.450615 10.1101/2021.06.30.450615 PMC8944272 35286197

[B21] Du W. Bautista J. F. Yang H. Diez-Sampedro A. You S.-A. Wang L. (2005). Calcium-sensitive potassium channelopathy in human epilepsy and paroxysmal movement disorder. *Nat. Genet.* 37 733–738. 10.1038/ng1585 15937479

[B22] Du X. Carvalho-de-Souza J. L. Wei C. Carrasquel-Ursulaez W. Lorenzo Y. Gonzalez N. (2020). Loss-of-function BK channel mutation causes impaired mitochondria and progressive cerebellar ataxia. *Proc. Natl. Acad. Sci. U.S.A.* 117:6023. 10.1073/pnas.1920008117 32132200 PMC7084159

[B23] Gandhi C. S. Isacoff E. Y. (2002). Molecular models of voltage sensing. *J. Gen. Physiol.* 120 455–463. 10.1085/jgp.20028678 12356848 PMC2229531

[B24] Geng Y. Deng Z. Zhang G. Budelli G. Butler A. Yuan P. (2020). Coupling of Ca(2+) and voltage activation in BK channels through the alphaB helix/voltage sensor interface. *Proc. Natl. Acad. Sci. U.S.A.* 117 14512–14521. 10.1073/pnas.1908183117 32513714 PMC7321994

[B25] Heim J. Vemuri A. Lewis S. Guida B. Troester M. Keros S. (2020). Cataplexy in patients harboring the KCNMA1 p.N999S mutation. *Mov. Disord. Clin. Pract.* 7 861–862. 10.1002/mdc3.13024 33043086 PMC7533972

[B26] Hite R. K. Tao X. MacKinnon R. (2017). Structural basis for gating the high-conductance Ca2+-activated K+ channel. *Nature* 541 52–57. 10.1038/nature20775 27974801 PMC5513477

[B27] Horrigan F. T. Aldrich R. W. (1999). Allosteric voltage gating of potassium channels II. Mslo channel gating charge movement in the absence of Ca2+. *J. Gen. Physiol.* 114 305–336. 10.1085/jgp.114.2.305 10436004 PMC2230644

[B28] Horrigan F. T. Aldrich R. W. (2002). Coupling between voltage sensor activation, Ca2+ binding and channel opening in large conductance (BK) potassium channels. *J. Gen. Physiol.* 120 267–305. 10.1085/jgp.20028605 12198087 PMC2229516

[B29] Horrigan F. T. Cui J. Aldrich R. W. (1999). Allosteric voltage gating of potassium channels I. Mslo ionic currents in the absence of Ca2+. *J. Gen. Physiol.* 114 277–304. 10.1085/jgp.114.2.277 10436003 PMC2230643

[B30] Hou P. Kang P. W. Kongmeneck A. D. Yang N. D. Liu Y. Shi J. (2020). Two-stage electro-mechanical coupling of a KV channel in voltage-dependent activation. *Nat. Commun.* 11:676. 10.1038/s41467-020-14406-w 32015334 PMC6997178

[B31] Hu L. Shi J. Ma Z. Krishnamoorthy G. Sieling F. Zhang G. (2003). Participation of the S4 voltage sensor in the Mg2+-dependent activation of large conductance (BK) K+ channels. *Proc. Natl. Acad. Sci. U.S.A.* 100 10488–10493. 10.1073/pnas.1834300100 12925732 PMC193588

[B32] Islas L. D. Sigworth F. J. (1999). Voltage sensitivity and gating charge in shaker and shab family potassium channels. *J. Gen. Physiol.* 114 723–742. 10.1085/jgp.114.5.723 10539976 PMC2230542

[B33] Javaherian A. D. Yusifov T. Pantazis A. Franklin S. Gandhi C. S. Olcese R. (2011). Metal-driven operation of the human large-conductance voltage- and Ca2+-dependent potassium channel (BK) gating ring apparatus. *J. Biol. Chem.* 286 20701–20709. 10.1074/jbc.M111.235234 21471215 PMC3121532

[B34] Jia Z. Yazdani M. Zhang G. Cui J. Chen J. (2018). Hydrophobic gating in BK channels. *Nat. Commun.* 9:3408. 10.1038/s41467-018-05970-3 30143620 PMC6109084

[B35] Jiang Y. Lee A. Chen J. Cadene M. Chait B. T. MacKinnon R. (2002a). Crystal structure and mechanism of a calcium-gated potassium channel. *Nature* 417 515–522. 10.1038/417515a 12037559

[B36] Jiang Y. Lee A. Chen J. Cadene M. Chait B. T. MacKinnon R. (2002b). The open pore conformation of potassium channels. *Nature* 417 523–526. 10.1038/417523a 12037560

[B37] Kaczorowski G. J. Garcia M. L. (2016). “Chapter twelve – developing molecular pharmacology of bk channels for therapeutic benefit,” in *International Review of Neurobiology*, ed. Contet C. (Cambridge, MA: Academic Press), 439–475. 10.1016/bs.irn.2016.02.013 27238271

[B38] Krishnamoorthy G. Shi J. Sept D. Cui J. (2005). The NH2 terminus of RCK1 domain regulates Ca2+-dependent BK(Ca) channel gating. *J. Gen. Physiol.* 126 227–241. 10.1085/jgp.200509321 16103277 PMC2266574

[B39] Lancaster B. Nicoll R. A. (1987). Properties of two calcium-activated hyperpolarizations in rat hippocampal neurones. *J. Physiol. (Lond.)* 389 187–203. 10.1113/jphysiol.1987.sp016653 2445972 PMC1192077

[B40] Latorre R. Oberhauser A. Labarca P. Alvarez O. (1989). Varieties of calcium-activated potassium channels. *Annu. Rev. Physiol.* 51 385–399. 10.1146/annurev.ph.51.030189.002125 2653189

[B41] Lee U. S. Cui J. (2009). {beta} subunit-specific modulations of BK channel function by a mutation associated with epilepsy and dyskinesia. *J. Physiol.* 587 1481–1498. 10.1113/jphysiol.2009.169243 19204046 PMC2678220

[B42] Lee U. S. Cui J. (2010). BK channel activation: structural and functional insights. *Trends Neurosci.* 33 415–423. 10.1016/j.tins.2010.06.004 20663573 PMC2929326

[B43] Li W. Aldrich R. W. (2004). Unique inner pore properties of BK channels revealed by quaternary ammonium block. *J. Gen. Physiol.* 124 43–57. 10.1085/jgp.200409067 15197222 PMC2229608

[B44] Li W. Aldrich R. W. (2006). State-dependent block of BK channels by synthesized shaker ball peptides. *J. Gen. Physiol.* 128 423–441. 10.1085/jgp.200609521 16966472 PMC2151574

[B45] Li X. Poschmann S. Chen Q. Fazeli W. Oundjian N. J. Snoeijen-Schouwenaars F. M. (2018). De novo BK channel variant causes epilepsy by affecting voltage gating but not Ca2+ sensitivity. *Eur. J. Hum. Genet.* 26 220–229. 10.1038/s41431-017-0073-3 29330545 PMC5839055

[B46] Liang L. Li X. Moutton S. Schrier Vergano S. A. Cogné B. Saint-Martin A. (2019). De novo loss-of-function KCNMA1 variants are associated with a new multiple malformation syndrome and a broad spectrum of developmental and neurological phenotypes. *Hum. Mol. Genet.* 28 2937–2951. 10.1093/hmg/ddz117 31152168 PMC6735855

[B47] Liu Y. Holmgren M. Jurman M. E. Yellen G. (1997). Gated access to the pore of a voltage-dependent K+ channel. *Neuron* 19 175–184. 10.1016/S0896-6273(00)80357-8 9247273

[B48] Long S. B. Campbell E. B. Mackinnon R. (2005a). Crystal structure of a mammalian voltage-dependent Shaker family K+ channel. *Science* 309 897–903. 10.1126/science.1116269 16002581

[B49] Long S. B. Campbell E. B. Mackinnon R. (2005b). Voltage sensor of Kv1.2: structural basis of electromechanical coupling. *Science* 309 903–908. 10.1126/science.1116270 16002579

[B50] Lorenzo-Ceballos Y. Carrasquel-Ursulaez W. Castillo K. Alvarez O. Latorre R. (2019). Calcium-driven regulation of voltage-sensing domains in BK channels. *eLife* 8:e44934. 10.7554/eLife.44934.018 PMC6763263 31509109

[B51] Lu Z. Klem A. M. Ramu Y. (2001). Ion conduction pore is conserved among potassium channels. *Nature* 413 809–813. 10.1038/35101535 11677598

[B52] Ma Z. M. Horrigan F. T. (2005). Voltage-sensing residues in S2 and S4 segments of the BK channel. *Biophys. J.* 88:100A.

[B53] Magidovich E. Yifrach O. (2004). Conserved gating hinge in ligand- and voltage-dependent K+ channels. *Biochemistry* 43 13242–13247. 10.1021/bi048377v 15491131

[B54] Mannuzzu L. M. Moronne M. M. Isacoff E. Y. (1996). Direct physical measure of conformational rearrangement underlying potassium channel gating. *Science* 271 213–216. 10.1126/science.271.5246.213 8539623

[B55] Marty A. (1981). Ca-dependent K channels with large unitary conductance in chromaffin cell membranes. *Nature* 291 497–500. 10.1038/291497a0 6262657

[B56] McManus O. B. Magleby K. L. (1991). Accounting for the Ca2+-dependent kinetics of single large-conductance Ca2+-activated K+ channels in rat skeletal muscle. *J. Physiol.* 443 739–777. 10.1113/jphysiol.1991.sp018861 1822543 PMC1179869

[B57] Miller J. P. Moldenhauer H. J. Keros S. Meredith A. L. (2021). Emerging spectrum of variants and clinical features in KCNMA1-linked channelopathy. *Channels* 15 447–464. 10.1080/19336950.2021.1938852 34224328 PMC8259716

[B58] Miranda P. Holmgren M. Giraldez T. (2018). Voltage-dependent dynamics of the BK channel cytosolic gating ring are coupled to the membrane-embedded voltage sensor. *eLife* 7:e40664. 10.7554/eLife.40664.014 PMC6301790 30526860

[B59] Moldenhauer H. J. Matychak K. K. Meredith A. L. (2020). Comparative gain-of-function effects of the KCNMA1-N999S mutation on human BK channel properties. *J. Neurophysiol.* 123 560–570. 10.1152/jn.00626.2019 31851553 PMC7052641

[B60] Niu X. Qian X. Magleby K. L. (2004). Linker-gating ring complex as passive spring and Ca2+-dependent machine for a voltage- and Ca2+-activated potassium channel. *Neuron* 42 745–756. 10.1016/j.neuron.2004.05.001 15182715

[B61] Pallotta B. S. Magleby K. L. Barrett J. N. (1981). Single channel recordings of Ca2+-activated K+ currents in rat muscle cell culture. *Nature* 293 471–474. 10.1038/293471a0 6273730

[B62] Pantazis A. Olcese R. (2012). Relative transmembrane segment rearrangements during BK channel activation resolved by structurally assigned fluorophore-quencher pairing. *J. Gen. Physiol.* 140 207–218. 10.1085/jgp.201210807 22802360 PMC3409098

[B63] Pantazis A. Gudzenko V. Savalli N. Sigg D. Olcese R. (2010a). Operation of the voltage sensor of a human voltage- and Ca^2+^-activated K^+^ channel. *Proc. Natl. Acad. Sci. U.S.A.* 107 4459–4464. 10.1073/pnas.0911959107 20160086 PMC2840143

[B64] Pantazis A. Kohanteb A. P. Olcese R. (2010b). Relative motion of transmembrane segments S0 and S4 during voltage sensor activation in the human BK(Ca) channel. *J. Gen. Physiol.* 136 645–657. 10.1085/jgp.201010503 21078868 PMC2995153

[B65] Pantazis A. Westerberg K. Althoff T. Abramson J. Olcese R. (2018). Harnessing photoinduced electron transfer to optically determine protein sub-nanoscale atomic distances. *Nat. Commun.* 9: 4738. 10.1038/s41467-018-07218-6 30413716 PMC6226468

[B66] Rockman M. E. Vouga A. G. Rothberg B. S. (2020). Molecular mechanism of BK channel activation by the smooth muscle relaxant NS11021. *J. Gen. Physiol.* 152:e201912506. 10.1085/jgp.201912506 32221543 PMC7266150

[B67] Sausbier M. Hu H. Arntz C. Feil S. Kamm S. Adelsberger H. (2004). Cerebellar ataxia and Purkinje cell dysfunction caused by Ca2+-activated K+ channel deficiency. *Proc. Natl. Acad. Sci. U.S.A.* 101 9474–9478. 10.1073/pnas.0401702101 15194823 PMC439001

[B68] Savalli N. Kondratiev A. Toro L. Olcese R. (2006). Voltage-dependent conformational changes in human Ca(2+)- and voltage-activated K(+) channel, revealed by voltage-clamp fluorometry. *Proc. Natl. Acad. Sci. U.S.A.* 103 12619–12624. 10.1073/pnas.0601176103 16895996 PMC1567928

[B69] Savalli N. Pantazis A. Yusifov T. Sigg D. Olcese R. (2012). The contribution of RCK domains to human BK channel allosteric activation. *J. Biol. Chem.* 287 21741–21750. 10.1074/jbc.M112.346171 22556415 PMC3381136

[B70] Schewe M. Sun H. Mert U. Mackenzie A. Pike A. C. W. Schulz F. (2019). A pharmacological master key mechanism that unlocks the selectivity filter gate in K(+) channels. *Science* 363 875–880. 10.1126/science.aav0569 30792303 PMC6982535

[B71] Schreiber M. Salkoff L. (1997). A novel calcium-sensing domain in the BK channel. *Biophys. J.* 73 1355–1363. 10.1016/S0006-3495(97)78168-2 9284303 PMC1181035

[B72] Seoh S. A. Sigg D. Papazian D. M. Bezanilla F. (1996). Voltage-sensing residues in the S2 and S4 segments of the Shaker K+ channel. *Neuron* 16 1159–1167. 10.1016/S0896-6273(00)80142-7 8663992

[B73] Shah M. M. (2014). Cortical HCN channels: function, trafficking and plasticity. *J. Physiol.* 592 2711–2719. 10.1113/jphysiol.2013.270058 24756635 PMC4104471

[B74] Shi J. Cui J. (2001). Intracellular Mg2+ enhances the function of BK-type Ca2+-activated K+ channels. *J. Gen. Physiol.* 118 589–606. 10.1085/jgp.118.5.589 11696614 PMC2233844

[B75] Shi J. Krishnamoorthy G. Yang Y. Hu L. Chaturvedi N. Harilal D. (2002). Mechanism of magnesium activation of calcium-activated potassium channels. *Nature* 418 876–880. 10.1038/nature00941 12192410

[B76] Storm J. F. (1987). Action potential repolarization and a fast after-hyperpolarization in rat hippocampal pyramidal cells. *J. Physiol. (Lond.)* 385 733–759. 10.1113/jphysiol.1987.sp016517 2443676 PMC1192370

[B77] Sweet T.-B. Cox D. H. (2008). Measurements of the BKCa channel’s high-affinity Ca2+ binding constants: effects of membrane voltage. *J. Gen. Physiol.* 132 491–505. 10.1085/jgp.200810094 18955592 PMC2571968

[B78] Tabarki B. AlMajhad N. AlHashem A. Shaheen R. Alkuraya F. S. (2016). Homozygous KCNMA1 mutation as a cause of cerebellar atrophy, developmental delay and seizures. *Hum. Genet.* 135 1295–1298. 10.1007/s00439-016-1726-y 27567911

[B79] Tang Q.-Y. Zeng X.-H. Lingle C. J. (2009). Closed-channel block of BK potassium channels by bbTBA requires partial activation. *J. Gen. Physiol.* 134 409–436. 10.1085/jgp.200910251 19858359 PMC2768800

[B80] Tao X. MacKinnon R. (2019). Molecular structures of the human Slo1 K(+) channel in complex with beta4. *eLife* 8:e51409. 10.7554/eLife.51409 31815672 PMC6934384

[B81] Tao X. Hite R. K. MacKinnon R. (2017). Cryo-EM structure of the open high-conductance Ca2+-activated K+ channel. *Nature* 541 46–51. 10.1038/nature20608 27974795 PMC5500982

[B82] Tian Y. Heinemann S. H. Hoshi T. (2019). Large-conductance Ca^2+^- and voltage-gated K^+^ channels form and break interactions with membrane lipids during each gating cycle. *Proc. Natl. Acad. Sci. U.S.A.* 116 8591–8596. 10.1073/pnas.1901381116 30967508 PMC6486743

[B83] Wang B. Rothberg B. S. Brenner R. (2009). Mechanism of increased BK channel activation from a channel mutation that causes epilepsy. *J. Gen. Physiol.* 133 283–294. 10.1085/jgp.200810141 19204188 PMC2654085

[B84] Wilkens C. M. Aldrich R. W. (2006). State-independent block of BK channels by an intracellular quaternary ammonium. *J. Gen. Physiol.* 128 347–364. 16940557 10.1085/jgp.200609579 PMC2151567

[B85] Wu Y. Yang Y. Ye S. Jiang Y. (2010). Structure of the gating ring from the human large-conductance Ca(2+)-gated K(+) channel. *Nature* 466 393–397. 20574420 10.1038/nature09252 PMC2910425

[B86] Xia X.-M. Zeng X. Lingle C. J. (2002). Multiple regulatory sites in large-conductance calcium-activated potassium channels. *Nature* 418 880–884. 12192411 10.1038/nature00956

[B87] Yang H. Hu L. Shi J. Delaloye K. Horrigan F. T. Cui J. (2007). Mg2+ mediates interaction between the voltage sensor and cytosolic domain to activate BK channels. *Proc. Natl. Acad. Sci. U.S.A.* 104 18270–18275. 17984060 10.1073/pnas.0705873104 PMC2084332

[B88] Yang H. Shi J. Zhang G. Yang J. Delaloye K. Cui J. (2008). Activation of Slo1 BK channels by Mg2+ coordinated between the voltage sensor and RCK1 domains. *Nat. Struct. Mol. Biol.* 15 1152–1159. 18931675 10.1038/nsmb.1507 PMC2579968

[B89] Yang J. Krishnamoorthy G. Saxena A. Zhang G. Shi J. Yang H. (2010). An epilepsy/dyskinesia-associated mutation enhances BK channel activation by potentiating Ca2+ sensing. *Neuron* 66 871–883. 20620873 10.1016/j.neuron.2010.05.009 PMC2907746

[B90] Yang J. Yang H. Sun X. Delaloye K. Yang X. Moller A. (2013). Interaction between residues in the Mg2+-binding site regulates BK channel activation. *J. Gen. Physiol.* 141 217–228. 23359284 10.1085/jgp.201210794 PMC3557308

[B91] Yazdani M. Zhang G. Jia Z. Shi J. Cui J. Chen J. (2020b). Aromatic interactions with membrane modulate human BK channel activation. *eLife* 9:e55571. 10.7554/eLife.55571 PMC7371421 32597752

[B92] Yazdani M. Jia Z. Chen J. (2020a). Hydrophobic dewetting in gating and regulation of transmembrane protein ion channels. *J. Chem. Phys.* 153: 110901. 10.1063/5.0017537 PMC9722268 32962356

[B93] Yesil G. Aralasmak A. Akyuz E. Icagasioglu D. Uygur Sahin T. Bayram Y. (2018). Expanding the phenotype of homozygous KCNMA1 mutations; dyskinesia, epilepsy, intellectual disability, cerebellar and corticospinal tract atrophy. *Balkan Med. J.* 35 336–339. 29545233 10.4274/balkanmedj.2017.0986 PMC6060973

[B94] Yuan P. Leonetti M. D. Hsiung Y. MacKinnon R. (2012). Open structure of the Ca2+ gating ring in the high-conductance Ca2+-activated K+ channel. *Nature* 481 94–97. 10.1038/nature10670 22139424 PMC3319005

[B95] Yuan P. Leonetti M. D. Pico A. R. Hsiung Y. MacKinnon R. (2010). Structure of the human BK channel Ca2+-activation apparatus at 3.0 A resolution. *Science* 329 182–186. 10.1126/science.1190414 20508092 PMC3022345

[B96] Yusifov T. Savalli N. Gandhi C. S. Ottolia M. Olcese R. (2008). The RCK2 domain of the human BKCa channel is a calcium sensor. *Proc. Natl. Acad. Sci. U.S.A.* 105 376–381. 10.1073/pnas.0705261105 18162557 PMC2224220

[B97] Zagotta W. N. Hoshi T. Aldrich R. W. (1994a). Shaker potassium channel gating. III: evaluation of kinetic models for activation. *J. Gen. Physiol.* 103 321–362. 10.1085/jgp.103.2.321 8189208 PMC2216839

[B98] Zagotta W. N. Hoshi T. Dittman J. Aldrich R. W. (1994b). Shaker potassium channel gating. II: transitions in the activation pathway. *J. Gen. Physiol.* 103 279–319. 10.1085/jgp.103.2.279 8189207 PMC2216838

[B99] Zeng X. H. Xia X. M. Lingle C. J. (2005). Divalent cation sensitivity of BK channel activation supports the existence of three distinct binding sites. *J. Gen. Physiol.* 125 273–286. 10.1085/jgp.200409239 15738049 PMC2234011

[B100] Zhang G. Geng Y. Jin Y. Shi J. McFarland K. Magleby K. L. (2017). Deletion of cytosolic gating ring decreases gate and voltage sensor coupling in BK channels. *J. Gen. Physiol.* 149 373–387. 10.1085/jgp.201611646 28196879 PMC5339509

[B101] Zhang G. Gibson R. A. McDonald M. Liang P. Kang P. W. Shi J. (2020). A gain-of-function mutation in KCNMA1 causes dystonia spells controlled with stimulant therapy. *Mov. Disord.* 35 1868–1873. 10.1002/mds.28138 32633875 PMC7572833

[B102] Zhang G. Huang S.-Y. Yang J. Shi J. Yang X. Moller A. (2010). Ion sensing in the RCK1 domain of BK channels. *Proc. Natl. Acad. Sci. U.S.A.* 107 18700–18705. 10.1073/pnas.1010124107 20937866 PMC2972974

[B103] Zhang G. Yang H. Liang H. Yang J. Shi J. McFarland K. (2014). A charged residue in S4 regulates coupling among the activation gate, voltage, and Ca2+ sensors in BK channels. *J. Neurosci.* 34 12280–12288. 10.1523/JNEUROSCI.1174-14.2014 25209270 PMC4160767

[B104] Zhang Z. B. Tian M. Q. Gao K. Jiang Y. W. Wu Y. (2015). De novo KCNMA1 mutations in children with early-onset paroxysmal dyskinesia and developmental delay. *Mov. Disord.* 30 1290–1292. 10.1002/mds.26216 26195193

[B105] Zhou Y. Xia X. M. Lingle C. J. (2020). functionally relevant site for paxilline inhibition of BK channels. *Proc. Natl. Acad. Sci. U.S.A.* 117 1021–1026. 10.1073/pnas.1912623117 31879339 PMC6969516

[B106] Zhou Y. Xia X.-M. Lingle C. J. (2011). Cysteine scanning and modification reveal major differences between BK channels and Kv channels in the inner pore region. *Proc. Natl. Acad. Sci. U.S.A.* 108 12161–12166. 10.1073/pnas.1104150108 21730134 PMC3141973

[B107] Zhou Y. Xia X.-M. Lingle C. J. (2015). Cadmium–cysteine coordination in the BK inner pore region and its structural and functional implications. *Proc. Natl. Acad. Sci. U.S.A.* 112:5237. 10.1073/pnas.1500953112 25848005 PMC4413310

